# Insights on Microbial Communities Inhabiting Non-Volcanic Hot Springs

**DOI:** 10.3390/ijms232012241

**Published:** 2022-10-13

**Authors:** Juan-José Escuder-Rodríguez, María-Eugenia DeCastro, Almudena Saavedra-Bouza, Manuel Becerra, María-Isabel González-Siso

**Affiliations:** Grupo EXPRELA, Centro de Investigacións Científicas Avanzadas (CICA), Departamento de Bioloxía, Facultade de Ciencias, Universidade da Coruña, 15071 A Coruña, Spain

**Keywords:** shotgun sequencing, aquificales, *Sulfurihydrogenibium*, rare biosphere, thermophiles, thermozymes

## Abstract

The northwest of Spain has an abundance of non-volcanic hot springs that, until recently, had only been used for thermalism activities. One of such hot springs, Muiño da Veiga, has now been explored using metagenomics to study the microbial community that inhabits these high-temperature circumneutral continental waters. Sequencing of the metagenome allowed the characterization of its composition, diversity, metabolic connections and potential as a source for thermozymes, as well as its ability to assemble MAGs. A diverse microbial community dominated by Bacteria domain members was revealed, particularly from the early-branching Aquificales group. The most abundant genus was *Sulfurihydrogenibium*, known for its implication in sulfur cycling and for forming mats that enable novel niches. The variety of primary producers with autotrophic pathways (and specifically the sulfur oxidizing pathway) expands the range of available nutrients, and the increase in biomass forms thicker mats, resulting in more available niches and broader microbial diversity. Nonetheless, certain metabolic pathways were attributed to less abundant members of the microbial community, reinforcing the idea that the rare biosphere plays important roles in the network of interactions present in an ecosystem and acts as genetic reservoirs. In addition, three of the assembled MAGs represent novel microbial diversity found in this hot spring. Moreover, the presence of enzymes and microorganisms with possible biotechnological applications was confirmed, including proteases, lipases and cell-wall degrading enzymes, pointing to the potential for the hot spring as a source for thermozymes.

## 1. Introduction

Since the first studies on microbial identification relying on phenotypical traits of pure cultures [1] and the notion of the great plate count anomaly [2], the importance of molecular methods for the classification of microorganisms is now fully accepted [3]. Cloning environmental DNA was deemed as a solution to microbial identification, but the technical requirements for assessing the metagenome were not met until the development of high-throughput sequencing technologies [1,4]. A wide range of platforms categorized as Next-Generation Sequencing (NGS) are available and have experienced several improvements overcoming some of the technical bottlenecks associated with obtaining sequencing data at a microbial community-level scale [4]. Depending on the strategy employed, there is a distinction between the cost-effective targeted sequencing of biomarker genes (in most studies, the variable regions of the 16S rRNA gene): one strategy focuses on answering the “Who are they?” biodiversity question, and the other is the shotgun metagenomic sequencing strategy that can answer the “What are they doing?” ecological question [5]. To analyze the enormous amount of data generated from NGS, an extensive and ever-growing number of bioinformatic tools have been developed [6]. Although metagenomics was first envisioned to explore the diversity of the soil microorganisms [7], it rapidly expanded to cover water environments, and in recent years, the role of the human-associated microbiome in regard to health and disease has become an increasingly important focus for research and has driven the development of many bioinformatic tools [8].

Due to their biotechnological potential and unique conditions that can resemble primitive Earth environments and even potential extra-terrestrial ecosystems, hot springs are expected to host microbial communities that hold high value to both basic and applied science [8]. Unlike many other well-studied hydrothermal systems, including Yellowstone National Park in the USA [9] and some regions in Iceland [10] and Japan [11], to mention a few; the hot springs in Ourense (Spain) have non-volcanic origins, with a circumneutral pH and moderately high temperature [9,10,11].

The geological setting of the region has been described previously [12,13]. In brief, it lies at the bottom of a hollow formed by the erosion process of Miño river and its two main tributaries. Two groups of granite crystalline rocks are contacted by intrusion along both sides of the Miño river, delimiting a north and a south side, all covered by alluvial soils. The granite rock presents almost no permeability, which makes the water flow only along faults and fractures. This in turn makes the water follow a deep circuit of percolation at higher elevations, storage and circulation with heat transfer through main fault systems [14] and emergence at lower elevations [12,13]. The process involves the interaction of water with minerals, adding solutes to its composition. As the water reaches greater depths, rock temperature increases and heat transfer takes place, explaining the higher occurrence of hot springs in the lower elevations of the valley, where water has circulated deeper and for longer periods of time. Aside from the high basal heat flow of the region, radioactive decay reactions in igneous rocks are also a possible additional source for heat transfer to the water [14]. One such hot spring in the region is the Muiño da Veiga site, located next to the Miño river, that up until recently has only been employed by humans for thermalism.

In the present study, we conducted a shotgun metagenomic sequencing study of the Muiño da Veiga hot spring metagenome in order to characterize the microbial community, its biotechnological potential and the main metabolic pathways involved in this thermophilic habitat. Moreover, the assembly of Metagenome-Assembled Genomes (MAGs) was performed, and up to 10 MAGs from the hot spring were identified taxonomically and functionally annotated. A diverse microbial community was found with several main metabolic pathways for the cycling of present elements. The community mostly comprised bacteria from the early-branching Aquificales group, and the potential as a source for thermozymes was confirmed. Some volcanic hot springs communities are predominated with the genus *Sulfurihydrogenibium* [9,10,11], which is the dominant genus in the microbial community of Muiño da Veiga as well. Nevertheless, Muiño da Veiga metagenome revealed high diversity and a low proportion of Archaea, highlighting specific characteristics of Aquificales-rich non-volcanic hot springs.

## 2. Results and Discussion

### 2.1. Assessment of the Microbial Community Potential for Retrieving Enzymes of Bio-Technological Interest

#### 2.1.1. Alignment against the SEED Subsystems Database

**Figure 1:**
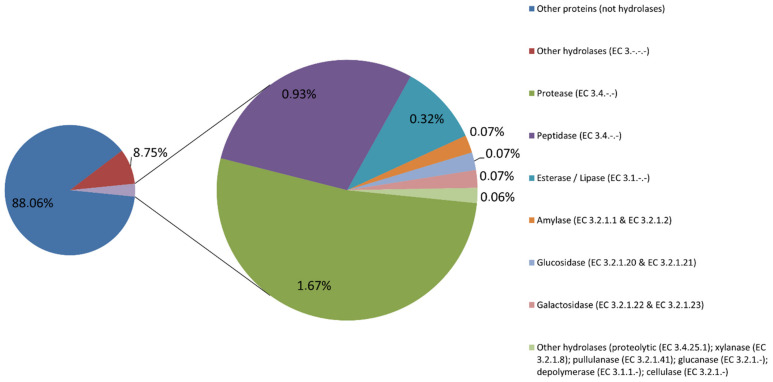
Relative abundances of hydrolases from the metagenomic sample obtained in Muiño da Veiga. Abundance was measured as the number of counts obtained by the DIAMOND aligner using a SEED subsystems database clustered using an identity of 98% (in order to reduce the size of the original database). Out of 30,132,990 counts, 88.06% were assigned to a subsystem function different from hydrolases; 8.75% were found to be hydrolases with no direct biotechnological applications in industry. The remaining 3.19% were found to be hydrolases with possible interest in industrial processes: proteases, peptidases, esterases and lipases, amylases, glucosidases, galactosidases, proteolytic enzymes, xylanases, pullulanases, glucanases, depolymerases and cellulases. Relative abundances of each enzyme family are shown along with their EC number.

We obtained a total of 30,132,990 counts, aligning to a reduced-size SEED subsystems database. We searched for enzymes that fall in the category of hydrolases (with Enzyme Commission (EC) number 3.-.-.-) and found 3,598,086 counts (11.94% of the total). We further examined these counts in order to find enzymes that have a potential interest as biotechnological products for various industrial applications. Such enzymes and their relative abundances are shown in Figure 1.

#### 2.1.2. Alignment to the Non-Redundant Database

**Figure 2:**
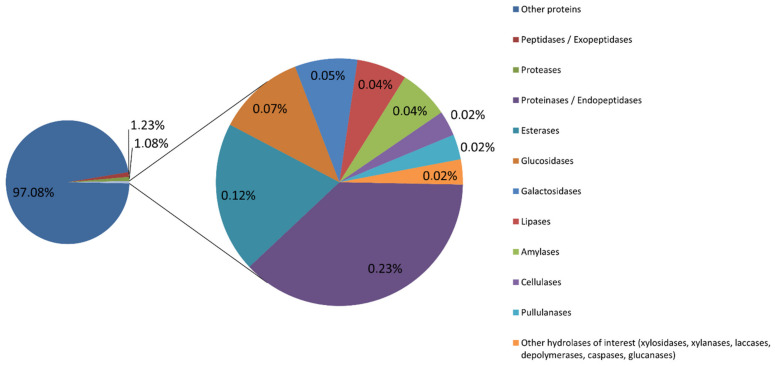
Relative abundances of hydrolases from the metagenomic sample obtained in Muiño da Veiga. Abundance was measured as number of counts obtained by the DIAMOND aligner using the NCBI NR database. The total amount of counts was 43,613,726. Of those, 1,276,574 counts (2.93%) were annotated as hydrolases with possible interest in industrial processes: proteases and peptidases (exopeptidases, endopeptidases and proteinases), esterases and lipases, amylases, cellulases, glucosidases, galactosidases, pullulanases, xylosidases, xylanases, laccases, depolymerases, caspases and glucanases. Relative abundances of each enzyme class are given as a percentage of the total number of counts obtained.

A total of 43,613,726 counts aligning to the non-redundant (NR) database were obtained. We performed a keyword-based search for enzymes that fall in the category of hydrolases with possible industrial interest, and 1,276,574 counts (2.93%) were selected. Figure 2 shows those enzymes and their relative abundances.

Our results showed that hydrolases were slightly over a tenth of the total proteins predicted by the bioinformatic analysis. Proteases and peptidases were the most abundant hydrolases, with potential industrial applications in both analyses. Although beyond the scope of the present study, the presence of such enzymes in the metagenome points towards the potential to retrieve thermozymes by conducting further bioprospecting studies.

### 2.2. MG-RAST Server Metagenome Overview

A summary of the metagenome statistics, as reported by the automated upload analysis of MG-RAST, is presented in Supplementary Table S1. These preliminary data show that even with the previous quality filtering of sequences, more than one-third of the sequences had to be further removed due to the quality criteria from the web-based analysis tool.

A small (6.64% of total sequences) portion of the sequences could not be associated to any feature deposited in the databases, and of the predicted features, 21.12% (19.72% of total sequences) were annotated as proteins of unknown function.

Sequencing depth was assessed by plotting the number of species (unique OTUs) found against the total number or reads needed to achieve them, with the rarefaction plot as the graphical representation (Supplementary Figure S1). Generally, good sequencing depth is achieved when adding more reads to the analysis had little effect on the diversity of species found, meaning that further sampling will have little effect on the diversity of species.

### 2.3. Metabolism and Interactions of the Microbial Community Inferred from the Metagenomic Analysis

**Figure 3:**
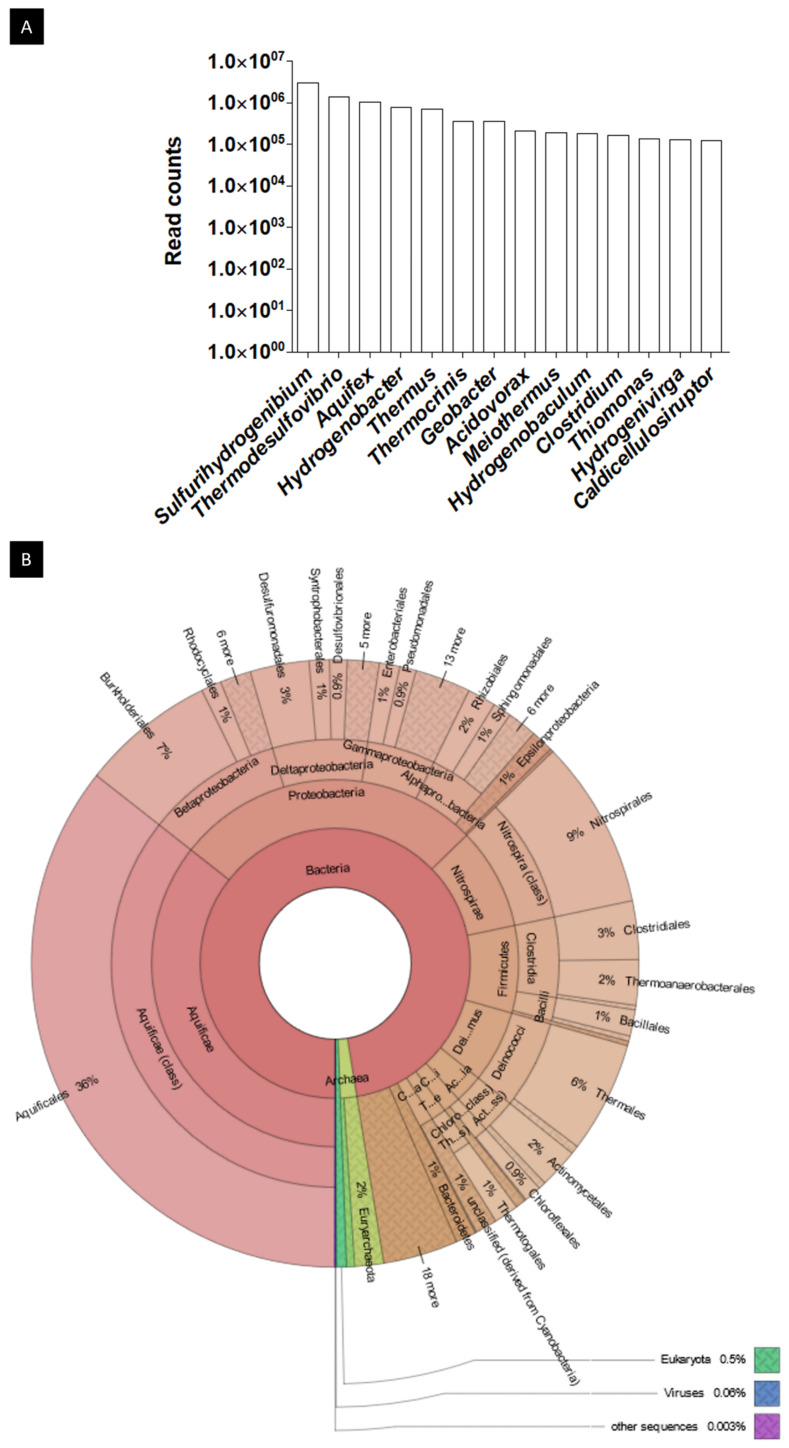
Results of the taxonomic read assessment for the Muiño da Veiga metagenome. (**A**) Rank of the top 14 most abundant genus in the metagenome, accounting for 56.2% of the total reads. The Y-axis represents in logarithm scale the number of reads for each genus. The genus outside this ranking accounted individually for less than 0.75% of the total reads, whereas the most abundant genus (*Sulfurihydrogenibium*) accounted for 19.3% of the total reads by itself, and the 8 most abundant genus accounted for more than half of the total reads. All the top-ranking genus belonged to the Bacteria domain. (**B**) Krona pie-chart representation of the taxonomical read assignments at the order level. Aquificales was the most abundant order with 36% of the reads, followed by Nitrospirales with 9% of the reads and Burkholderiales with 7%. Bacteria was the dominant domain with 97% of the reads, Archaea represented only 2% of the reads and the remaining reads belonged to Eukaryota, viruses and unassigned sequences.

An in-depth analysis conducted in the MG-RAST server was employed to describe the microbial community’s composition in the hot spring and to infer metabolic pathways that may explain the complex network of interactions between microorganisms inhabiting it. For the RefSeq database, a total of 15,369,172 hits were obtained for the taxonomical assessment of reads, whereas the KEGG Orthology (KO) database returned 7,135,862 hits and Subsystems 6,223,410 both for the functional assessment of reads. Figure 3 shows the most abundant genera of the hot spring (that covered more than 50% of the total reads) found by alignment to the RefSeq database and the percentages of each taxonomical category at the order level represented using the KRONA web visualization tool.

**Figure 4:**
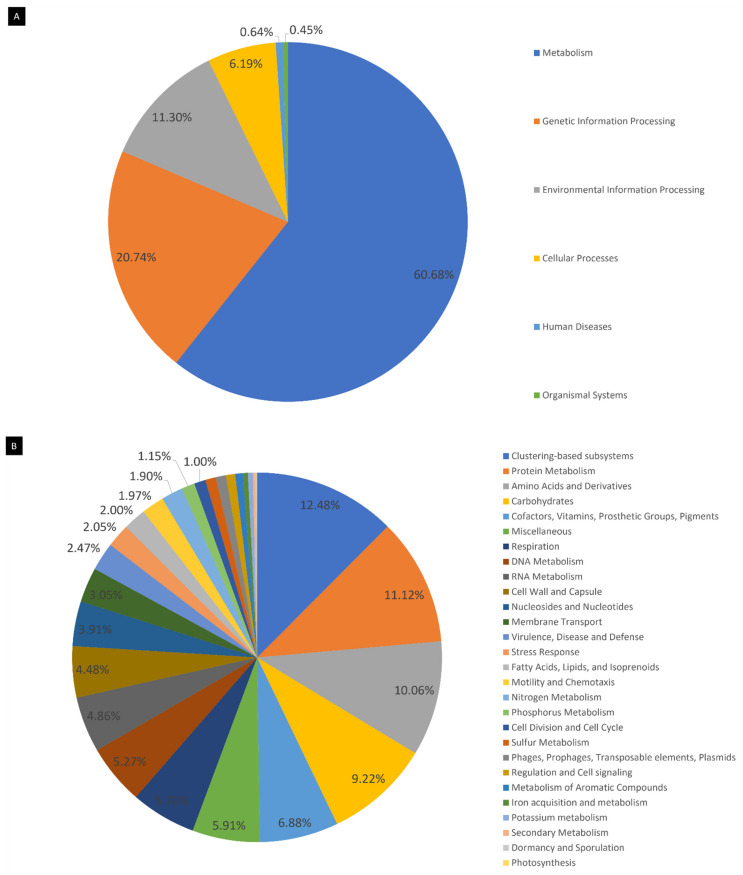
Pie charts of the functional categorization of reads. (**A**) KO database alignment; 60.7% of the features were assigned to the Metabolism category, 20.7% to Genetic Information Processing and 11.3% to Environmental Information Processing. (**B**) Subsystems database alignment; 12.5% of the features were categorized as Clustering-based subsystems, 11.1% as Protein Metabolism, 10.1% as Amino Acids and Derivatives, 9.2% as Carbohydrates, 6.9% as Cofactors, Vitamins, Prosthetic Groups and Pigments, 5.9% were assigned to the Miscellaneous category and 5.7% to Respiration.

Alignment to the KO and Subsystems databases allowed the function assignment of sequences. Pathways from the Kyoto Encyclopedia of Genes and Genomes (KEGG) were reconstructed, and metabolic routes were predicted to understand which ones were involved in the thermophilic microbial community, making possible the assessment of possible microbial interactions within the hot spring ecosystem. It is worth noting that this information ought to be supplemented with additional data published in the literature on the most abundant genera, as sequence information alone is not considered enough for fully describing such interactions and for fully contextualizing the complex metabolic network of a extremophilic microbial community [15]. Moreover, the same need has been established for rare microorganisms of a microbial community, as metagenomics alone is deemed to not always be sufficient for assessing the full metabolic potential of the so-called rare biosphere [16]. In this regard, an extensive search for the dominant and rare genera data on metabolism and core genomes and pangenomes was conducted to further understand complex metabolic interactions in the Muiño da Veiga hot spring ecosystem, keeping in mind that these inferences are inherently biased [17], but nevertheless offer a framework that can be combined with the functional assessment of reads for putative descriptions. The breakdown of reads assigned to functional categories in each database is represented in Figure 4.

**Figure 5:**
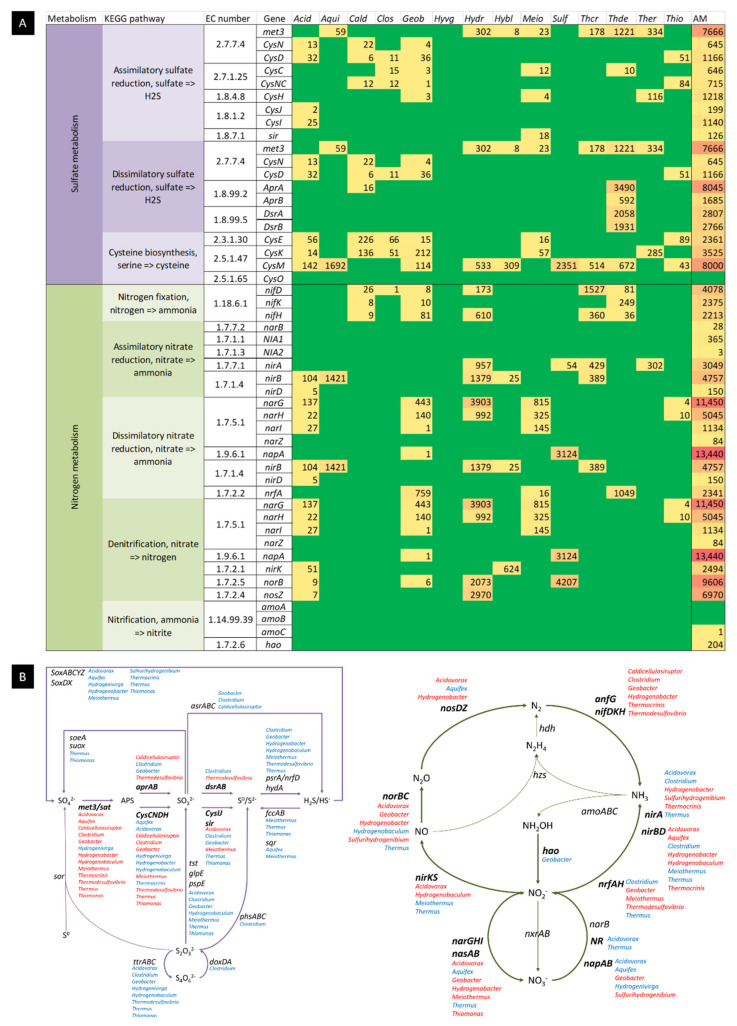
Metabolic assessment of the Muiño da Veiga metagenome in main genera of the microbial community. (**A**) The occurrence of genes in the metagenome from Muiño da Veiga involved in selected metabolic processes in main members of the microbial community as found by cross referencing KO and RefSeq databases. In green, genes that returned zero reads. In yellow to red gradient, the number of reads (in ascending order) that have a hit both KO and RefSeq databases. *Acid*: *Acidovorax*; *Aqui*: *Aquifex*; *Cald*: *Caldicellulosiruptor*; *Clos*: *Clostridium*; *Geo*: *Geobacter*; *Hyvg*: *Hydrogenivirga*; *Hydr*: *Hydrogenobacter*; *Hybl*: *Hydrogenobaculum*; *Meio*: *Meiothermus*; *Sulf*: *Sulfurihydrogenibium*; *Thcr*: *Thermocrinis*; *Thde*: *Thermodesulfovibrio*; *Ther*: *Thermus*; *Thio*: *Thiomonas*; AM: all reads across the metagenome. (**B**) Sulfur and nitrogen cycles in the Muiño da Veiga metagenome. Names in bold represent genes that were present in the cross reference of the KO and RefSeq databases (our data) with the metagenome for the main genus of the community. Thick lines represent reactions that were supported by either the cross reference of KO and RefSeq databases with the metagenome or by cross reference of the Uniprot database for the Enzymatic function (EC number) and the Organism name (genera) of the main genus of the community (published data). Genus names in red were present in the cross reference between the KO and the RefSeq databases, and genus names in blue were present in the cross refence of the Uniprot database for the enzymatic function (EC number) and the organism’s name (genera).

The occurrence of genes related to sulphur and nitrogen metabolisms and the network of metabolic interactions in both cycles for the Muiño da Veiga hot spring community is provided in Figure 5.

#### 2.3.1. Dominant Genera and Main Metabolic Pathways of the Muiño da Veiga Hot Spring Microbial Community

The presence of several sulphur utilizing genera as part of the dominant genera of the microbial community pointed to the importance of the role of this metabolic pathway in the ecosystem. Indeed, the dominant genus was *Sulfurihydrogenibium* (19.3% of total reads), which contains neutrophilic, thermophilic, and anaerobic to microaerobic species [18]. Species in this genus are chemolitoautotrophs and facultatively heterotrophs. They are able to employ sulphur compounds as electron donors and molecular oxygen as the electron acceptor, but some species are known to use other donors (such as molecular hydrogen) and acceptors (nitrate, Fe(III) or arsenate, for example) as well [19]. We also found *Thermodesulfovibrio* (8.78%), *Aquifex* (6.60%), *Hydrogenobacter* (4.92%), *Thermus* (4.64%), *Thermocrinis* (2.33%), *Geobacter* (2.33%), *Hydrogenobaculum* (1.15%), *Thiomonas* (0.90%) and *Hydrogenivirga* (0.83%) as prevalent genera related to this metabolism. Sulphur metabolism was also confirmed to be present, as revealed by the KEGG mapping assessment, as several key enzymes for the reduction in the fixation of this element had assigned reads by alignment to the KO and Subsystems databases (Figure 5). *Sulfurihydrogenibium*, *Aquifex*, *Hydrogenobacter*, *Thermocrinis*, *Hydrogenobaculum*, *Thiomonas* and *Hydrogenivirga* are sulphur-oxidizing (sulphur compounds are used as electron donors), whereas *Thermodesulfovibrio* and some *Thermus* and *Geobacter* species have a sulfate-reducing metabolism (sulphur compounds act as the electron acceptor). Whereas a synergetic cycling of the sulphur compounds is expected to occur between the two groups, each member within each group will have to fill a certain niche or enter competition when very similar metabolisms are present.

#### 2.3.2. Sulphur Oxidizing Bacteria

As mentioned, *Sulfurihydrogenibium* dominates the hot spring, with almost one-fifth of the total reads assigned to this genus. Their plasticity, acting as anaerobes or microaerobes and as chemolitoautotrophs or facultatively heterotrophs, probably contributes to their success in the Muiño da Veiga hot spring as it makes them adaptable to small disturbances in environmental conditions. Hot springs where *Sulfurihydrogenibium* dominates over other microorganisms have been reported, although our results show a more diverse community than other hot springs [20,21], which may be attributed to a process of adaptation to changing conditions in the hot spring. Moreover, Muiño da Veiga has rather neutral pH and temperatures (pH 7 and 68 °C) that are not in the hyperthermophilic range and can be considered more favourable to more varied communities than acidic hot springs or those with very high temperatures [17]. Nevertheless, the Aquificae group (to which abundant genera *Sulfurihydrogenibium*, *Aquifex*, *Hydrogenobacter*, *Thermocrinis*, *Hydrogenobaculum* and *Hydrogenivirga* belong to) has been highlighted as the main carbon fixator in terrestrial hot springs, with its energy metabolism driven by either hydrogen or reduced sulphur compound oxidation [21].

The microaerophile and strictly chemolithoautotrophic genus *Aquifex* uses hydrogen, thiosulfate and elemental sulphur as electron donors and oxygen and nitrate as electron acceptors [22]. They fixate all necessary carbon from environmental CO_2_ using the reverse (reductive) Tricarboxylic Acid Cycle (TCA) and perform gluconeogenesis using the the Embden–Meyerhof–Parnas pathway [23]. As they have an anaerobic anabolism while displaying an aerobic catabolism, they are deemed primitive microorganisms. Their microaerobic growth conditions are explained by this duality in their metabolism [24]. Very similarly, the *Hydrogenobacter* genus is microaerophilic and obligately chemolithotrophic. They have a respiratory metabolism using O_2_ as the electron acceptor and molecular hydrogen as the electron donor, but reduced sulphur compounds can also be used [25,26]. Carbon dioxide is fixed through the TCA cycle [27], and ammonium and nitrate salts serve as nitrogen sources [28]. As with *Aquifex*, this genus has an ambivalent metabolism of anaerobic anabolism and aerobic catabolism, supporting their position in the early-branching Aquificales order [29]. Their very similar metabolisms may suggest competence for the same metabolic niche within the community.

*Thermocrinis* is a genus of microaerophilic thermophiles with chemolithoautotrophic or chemoorganoheterotrophic growth [30]. For chemolithoautotrophic growth, all species can use molecular hydrogen, thiosulfate and elemental sulphur as sole electron donors and oxygen acting as an electron acceptor [31,32]. It has also been discovered that some species can use arsenite and monothioarsenate as electron donors that produce arsenate, adding metabolic diversity to the microbial community [33]. Chemoorganoheterotrophic growth is more varied, where simple or complex organic compounds can serve as carbon sources for some species, while others lack such capability [34]. The genus has often been an abundant representative of microbial communities in terrestrial hot spring ecosystems [31].

*Hydrogenobaculum* is a genus with respiratory metabolism and is more commonly associated with acidic hot springs [35]. They can use molecular hydrogen and reduced sulphur compounds as electron donors and molecular oxygen as electron acceptor, whereas carbon dioxide can be the sole carbon source that is fixed following the reductive tricarboxylic acid cycle [36]. Arsenite is known to be oxidized to arsenate in the absence of H_2_S in some species, but it is not used as an energy source [37,38], and this is likely because sulphide can inhibit microbial As(III) oxidases [39]. The dominance of this genus in certain hot springs has been explained by their ability to adapt to changes in temperature and oxygen concentrations [15].

*Thiomonas* comprises a genus of acidophilic obligate aerobes, with some species moderately thermophilic. They are facultative chemolithoautotrophs that are unable to denitrify nor oxidize ferrous iron. They can use a range of complex organic compounds and sugars as carbon sources or carbon dioxide through the Calvin–Benson–Bassham cycle, using reduced sulphur compounds, such as thiosulfate, or elemental sulphur as electron donors [40]. As heterotrophs, they can grow on acetate or pyruvate, but molecular hydrogen has been reported to be an electron donor in some strains as well [41]. It has been reported that members of this genus are important As(III)-oxidizing microorganisms in arsenic-contaminated ecosystems, removing this more toxic arsenic species coupled to their energy metabolism, with key metabolic genes such as those involved in CO_2_ fixation regulated differently between strains [42].

Members of *Hydrogenivirga* are anaerobic to microaerobic thermophilic microorganisms with a strict NaCl requirement for growth. They are strictly chemolithoautotrophic, with the ability to use molecular hydrogen or elemental sulphur as electron donors and oxygen or nitrate as electron acceptors [43,44].

#### 2.3.3. Sulphur Reducing Bacteria

Both *Thermodesulfovibrio* [45] and *Geobacter* are strict anaerobes and both can undergo the reduction of sulphur compounds and Fe(III), but nitrate is also a possible electron acceptor for species in both genera [45,46]. The coupling of acetate oxidation to the reduction of either Fe(III) or elemental sulphur creates a link in iron and sulphur geochemistry in anaerobic conditions [47]. It is worth mentioning that *Geobacter* is considered a mesophilic genus. While the presence of non-thermophilic genera in the hot spring is surprising at first, studies of the genome structure revealed a number of heat-shock proteins in some non-thermophilic strains, that could explain their presence in habitats with a higher temperature than their optima [48]. Interestingly, there are species within the genus *Thermodesulfovibrio* that use hydrogenotrophic methanogens as the electron-accepting system [49], which may be of relevance because of the presence of methanogens in the rare genera portion of the community, as discussed further below.

On the other hand, the genus *Thermus* is considered one of the most metabolically varied within thermophilic microorganisms inhabiting hot springs and, thus, is less specialized than the other two sulphur-reducing bacteria. Although some species are obligate aerobes, an analysis of the pangenome reveals anaerobic respiration capabilities, with species capable of polysulfide, ferric iron and nitrate reduction, in a decreasing frequency order, and the role of the genus in the associated metabolic pathways seems to be more relevant than first thought [50,51]. The oxidation of pyruvate, lactate and acetate as electron donors coupled to Fe(III) reduction is documented [51]. Metal-reducing species of *Thermus* are thought to be widely distributed due to the broad range of electron acceptors available through their metabolism [51].

#### 2.3.4. Non-Sulphur Metabolism-Related Microorganisms within the Dominant Genera of the Muiño da Veiga Hot Spring

Although most of the most abundant genera in the hot spring could be linked to various degrees to the sulphur cycle pathways, there were also several of them that did not have described species involved in this metabolism. As a common trait for these microorganisms not involved in the sulphur cycle, we found that all were specialized in the use of various simple to complex carbohydrates and, in most instances, had some degree of involvement in the nitrogen cycle. Their success as prominent members of the hot spring probably comes from this divergence from the apparent main metabolic pathways involved in the microbial community, as these species probably are filling niches that complement the use of nutrients and energy sources that are not exploited otherwise.

*Acidovorax* (1.34%) is a reclassified genus from *Pseudomonas* with at least 15 different species [52], with environmental species (from soil and freshwater) and phytopathogenic species. In water, they are often associated with blooming populations of cyanobacteria. Although first described as mesophilic species, several studies across the world have now linked some *Acidovorax* species to moderate [53,54] to high temperature hot springs [55,56] and, in particular, to acid-sulphate springs or iron-rich springs. The presence of temperature-resistant proteins has been reported and explains their ability to grow in thermophilic habitats [56]. They are aerobic and chemoorganotrophic, using oxygen as the terminal electron acceptor, or in some species using the heterotrophic denitrification of nitrate metabolic pathway [52,57]. Their metabolism of carbohydrates is expected to complement other species enzymatic pathways [53], for example, with the use of benzoate and xylene degradation.

The genus *Clostridium* (1.07%) is composed of a group of heterogenous obligate anaerobic species, and it is very widely distributed. They are known for their genetic plasticity, which enables species in the genus to use a wide range of nutrients and to colonize various habitats, including hot springs. The identification of *Clostridium* pangenome core genes points out that they can use glucose and glycerol sole carbon sources and that ammonium is the preferred source of inorganic nitrogen [58]. Several thermophilic species have been described, with biotechnologically interesting metabolic capabilities such as the ability to degrade cellulose and the production of bioethanol by the fermentation of cellulose and hemi-cellulose degradation-derived compounds [59], which also hints at the metabolic niche that they fulfil in the community.

Members of *Caldicellulosiruptor* (0.78%) are strict thermophilic anaerobes. Their metabolism consists in the fermentation of monosaccharides, disaccharides and polysaccharides as carbon sources, including cellulose as the sole carbon source, which makes them attractive from a bioprospecting standpoint [60]. All species are capable of glycolysis through the Embden–Meyerhof–Parnas pathway, the fermentation of xylose following the non-oxidative pentose phosphate pathway and the reduction of pyruvate with an incomplete TCA cycle [61].

#### 2.3.5. Nitrogen Metabolism

As mentioned when discussing their respective metabolisms, several predominant genera are also involved in the nitrogen cycling of the hot spring. *Sulfurihydrogenibium*, *Aquifex*, *Hidrogenivirga*, *Thermodesulfovibrio* and *Geobacter*, and some *Thermus*, *Acidovorax* and *Meiothermus* species are all reported to use nitrate as an electron acceptor and *Hydrogenobacter* and *Clostridium* grow on ammonium or nitrate salts as nitrogen sources. KEGG mapping allowed the reconstruction of the main metabolic reactions in the nitrogen cycle, with all major enzymes present for all pathways except for anaerobic ammonium oxidation (anammox), which was completely absent. The pathways present included dissimilatory and assimilatory nitrate reduction, denitrification, nitrogen fixation and complete nitrification.

The most abundant genus of the hot spring, *Sulfurydrogenibium*, is known to be able to perform the complete denitrification of nitrate to molecular nitrogen [18]. *Acidovorax* is known to also perform denitrification [52,57], and main genes involved in this process such as *nirK*, *norB* and *noZ* could all be linked to *Acidovorax* in our metagenomic data by cross referencing KO and RefSeq databases, an association supported by previously reported data [62]. The denitrification of nitrate to form nitrite and molecular nitrogen is also described in *Aquifex* [22] and *Hydrogenivirga* [44].

*Hydrogenobacter* species are able to perform nitrate reduction [63], and we found the *nirA* gene when we cross referenced functional and taxonomical databases.

Regarding nitrogen fixation, the *nifH* gene for nitrogenase was found in the KO database alignment to the metagenome, and species from the genera *Thermodesulfovibrio*, *Thermocrinis*, *Hydrogenobacter* and *Caldicellulosiruptor* are all reported to possess the *nifH* gene and are putative nitrogen-fixing microorganisms [64,65]. A cross reference between the KO database and the RefSeq database confirmed all four genera harbouring the *nifH* gene in the Muiño da Veiga metagenome.

Lastly, the oxidation of ammonia to nitrate through the nitrification pathway (through *amoABC*, *hao* and *nxrAB*/*narGH* genes) was found to be present (Figure 5). A single hit was found against the KO database for the *amoC* gene that cross referenced with the RefSeq database as a *Nitrosospira* genus bacterium hit, a rare member of the microbial community (0.13%). Moreover, the archaeal *amoA*-harbouring genus *Nitrosopumilus* [66] was also present in the metagenome, although it was present as a very rare member of the ecosystem (0.01%), opposite to other reports where ammonia oxidizing archaea had been described as playing a dominant role in the nitrogen cycle in a large number of terrestrial hot springs [67,68]. The *hao* gene was present in the metagenome, with hits only from the Deltaproteobacteria class, corresponding to relatively low abundant genera in the hot spring *Anaeromyxobacter* (0.35%) and *Desulfobacterium* (0.06%). Both *narG* and *narH* (but not *nxrA* nor *nxrB*) were present, as revealed by alignment to the KO database, and by cross referencing with the RefSeq database, main genera from the hot spring, including *Hydrogenobacter*, *Meiothermus*, *Geobacter* and *Acidovorax*, were revealed to harbour it. As only the last step of nitrification was represented by dominant genera of the ecosystem, the relevance of nitrite conversion to nitrate is probably more significant in the hot spring than complete nitrification from ammonia, which may be used by other microorganisms as a nitrogen source rather than an energy source in the nitrogen cycle.

#### 2.3.6. Hydrogen Metabolism

Hydrogen plays an important role in some hot springs communities as it links several metabolic pathways, especially as a key intermediate in anaerobic conditions [69]. For example, its generation by nitrogen-fixing bacteria or by fermentative metabolism, as well as its role as an electron donor and its consumption by sulphate-reducing bacteria and methanogens in hot springs, is well documented [69,70]. However, its importance in the metabolism is dependent of several factors (availability compared to other electron donors, the presence of coupling electron acceptors and how thermodynamically favourable the coupling is compared to others) [71,72]. Reportedly acidic hot springs are generally more prone to have hydrogen-favoured metabolisms compared to alkaline ones where sulphur compounds may be preferred [71]. A considerable number of dehydrogenases were detected when reads were aligned to the KO functional database. However, dehydrogenases constitute an heterogenous group of enzymes that are hard to classify and make functional predictions based on the sequence alone [71]. Nevertheless, it is noteworthy that several of the dominant genera of the hot spring contain potential hydrogen-using species, as molecular hydrogen can be used as an electron donor by *Sulfurihydrogenibium*, the dominant genus of the hot spring. The same is true for all other sulphur-oxidizing genera prevalent in the hot spring: *Aquifex*, *Hydrogenobacter*, *Thermocrinis*, *Hydrogenobaculum*, some *Thiomonas* species and *Hydrogenivirga*.

#### 2.3.7. Carbon Metabolism

KEGG mapping using the KO database revealed the dissimilatory metabolism of carbon, including a complete TCA cycle and almost complete Gluconeogenesis and Pentose Phosphate pathways in the metagenome. For assimilatory pathways, a complete reverse TCA cycle and an almost complete reverse Pentose Phosphate cycle (Calvin cycle) were also found. These results made apparent that main pathways for the use of carbon were all present in the microbial community. Given the results from the main genera survey with the RefSeq database, it seems likely that the reverse TCA cycle for CO_2_ fixation is a main driving force in the metabolic network of the Muiño da Veiga ecosystem (as mentioned before, Aquificae may constitute the main carbon fixators in terrestrial hot springs [21]. Nevertheless, the range of substrates that can be used by the community inferred from the metabolic potential of the dominant genera in the metagenome is remarkable, as described in the genera descriptions above. With simple to complex compounds as possible substrates, including (as an example) complex carbohydrates such as cellulose and xylan, the potential for this habitat as a source of interesting metabolites from a biotechnological standpoint is highlighted.

#### 2.3.8. Rare Species Metabolism

The so-called rare biosphere is often defined by an arbitrary numerical threshold of sequence counts (often around 0.1% to 0.01% relative abundance in a data set) [73]. Although classically ignored in favour of the dominant genera in a microbial community, it is now widely accepted that the rare members of the community can play uneven key roles in metabolic pathway networks such as element cycling and bioremediation [74] and that they constitute a genetic and functional diversity bank that supports ecological resilience [73,74]. Moreover, the composition of the rare fraction of a microbial community is expected to be highly variable in the event of changes in the ecosystem environmental conditions [74], or in other words, some species might be only conditionally or temporary rare and alterations in the ecosystem would promote such species to more prominent ecological roles [16,74]. Two particular metabolic processes were found by KEGG mapping using the KO database that could only be attributed to genera that could be regarded as rare, namely photosynthesis and methanogenesis.

##### Photosynthesis in Extreme Temperature Conditions

Almost complete Photosynthesis (00195) and Carbon Fixation in Photosynthetic microorganisms (00710) KEGG mapping pathways were present in the Muiño da Veiga metagenome, indicating the possibility of this autotrophic metabolism playing a role in the microbial community. Indeed, photosynthesis is possible at the temperature of the hot spring (68 °C) as the upper temperature limit is considered 73 °C in non-acidic habitats [75]. Some species of the Cyanobacteria phyla and a limited number of Chloroflexi ones have been observed to play the role of primary production through the photosynthetic pathway in hot springs, especially in alkaline conditions, where temperature plays an important role in species diversity [76]. We found that the major genera responsible for photosynthesis in hot springs were present in the metagenome, although their abundance was somewhat low compared to other much prominent members of the microbial community, which was also in agreement to the KEGG mapping often returning singular hits for specific enzymes involved in the photosynthetic pathway. For Cyanobacteria, we found both *Synechoccus* (0.23% of total reads) and *Thermosynechococcus* (0.06%) present in the sample, as well as photosyntetic Cloroflexi [76] *Roseiflexus* (0.56%), *Chloroflexus* (0.31%) and *Oscillochloris* (0.05%). The reads belonging to these major genera of photosynthetic microorganisms related to hot springs represented 1.21% of the total sequences. Moreover, the subterranean nature of these waters limits their exposition to sunlight (Supplementary Figure S1), although the surface of the hot springs could sustain the growth of microorganisms that do use these metabolic pathways. Taking it all together, photosynthesis probably plays a very minor role compared to other autotrophic metabolic routes that are also present in the metagenome and related to more abundant genera.

##### Archaea and Roles in Methane Metabolism

Several different Archaea genera were found in the metagenome, representing a small fraction of the total reads of the metagenome (1.96%). Of the total assigned Archaea reads, 57.15% aligned with nine predominant genera, many of which were found to be strict anaerobes and methanogens mainly belonging to the *Euryarchaeota* phylum, with the exception of the genus *Metallosphaera* from the *Crenarchaeota* phylum. Indeed, the Methane Metabolism pathway was mostly complete by KEGG mapping of the metagenomic reads, including all four of the methanogenesis modules, although the aceticlastic fermentation of acetate (acetate decarboxylation) returned more hits than the others, and the catabolism of methylamines was the most incomplete.

Nevertheless, the most abundant Archaea genus was *Methanosarcina* (0.20%), which is considered one of the most diverse methanogenic archaea regarding their metabolism. These anaerobic thermophiles can use all known methanogenic substrates using the four different catabolic pathways (CO_2_ reduction with H_2_, methyl reduction with H_2_, aceticlastic fermentation of acetate or methylotrophic catabolism of methanol, methylated amines and dimethylsulfide), and some species can grow without methane production using carbon monoxide; moreover, most species can fix molecular nitrogen [77]. Other thermophilic anaerobic methanogens ranking as the most abundant Archaea included *Methanocaldococcus* (0.14%) and *Methanococcus* (0.11%) [78,79], *Methanosaeta* (*Methanotrix*) (0.10%) that follows the acetoclastic (acetate decarboxylation) pathway for metanogenesis [80,81] and *Methanothermobacter* (0.8%), which is capable of reducing CO_2_ to methane by using H_2_ (and some species of formate as well) as electron donor. [82] Notably, we found the thermophilic, anaerobic and methanogenic *Archeoglobus* [83] (0.16%) as the second most abundant Archaea genus. This genus includes species that grow as chemolithoautotrophs in the presence of H_2_, CO_2_ and thiosulfate and as chemoorganotrophs with several complex organic compounds, using sulfate, sulfite and thiosulfate as electron acceptors.

Other non-methanogenic genera within the most abundant archaea included *Pyrococcus* (0.13%) and *Thermococcus* (0.11%), hyperthermophilic anaerobes that grow as chemoorganoheterotrophs capable of the fermentation of complex organic substrates and reducing sulphur species [84,85]. Lastly, *Methallosphaera* (0.09%) was the only aerobic archaea found within the nine dominant genera, which contains facultatively chemolithoautotrophic species that are capable of growing on sulfidic ores or elemental sulphur or chemoorganotrophically using complex organic substrates but not sugars [86].

With the exception of the methanogenic species, the fact that the metabolism characteristics of Archaea species are similar to those of the predominant bacteria genera induces their competition for similar resources in the hot spring, serving as a putative explanation for the lesser occurrence of these other species in the community.

### 2.4. Assembly of MAGs, Taxonomic Classification, and Functional Annotation

#### 2.4.1. KBase Pipeline Read Processing

FastQC flagged duplicate content on the 104,080,624 raw reads, an uneven base distribution towards the right end of the sequences and a high percentage of unassigned (N) bases in the same region. The trimmomatic output produced 73,790,737 reads (70.9%) from both forward and reverse files that passed the quality filter. The PrinSeq removal of low complexity reads resulted in 73,239,539 total reads. The second run of FastQC revealed an overall improvement in sequence quality and the full removal of Ns in the reads file. After the assembly of the reads into contigs using metaSPAdes, the quality was assessed with QUAST. A total of 13,007 contigs were generated with a N50 of 9564. MetaBAT2 was able to bin 7602 contigs into 49 bins. Lastly, CheckM was used to select the actual MAGs from the total bins, identifying 10 MAGs (bin002, bin008, bin010, bin014, bin016, bin019, bin023, bin026, bin042 and bin043) for their completeness and their contamination rates. The report from CheckM is provided in Supplementary Figure S3 and Supplementary Table S2.

#### 2.4.2. MAGs Analysis

MAGs were classified taxonomically using GTDB-Tk, which uses a combination of placements in a phylogenetic tree (topology), Average Nucleotide Identity (ANI) and the Relative Evolutionary Distance (RED). Results for the taxonomic assignment are presented in Table 1.

Most (40%) MAGs correspond to the Aquificae class (MAG008, MAG023, MAG026 and MAG043). Genera previously found to be most abundant in the hot spring were also identified in the MAG analysis, notably *Sulfurihydrogenibium* (MAG023)*, Thermodesulfovibrio* (MAG042) and *Hydrogenobacter* (MAG008). Two MAGS (MAG016 and MAG 043) could not be assigned to a genus and may represent taxonomic novelty found in the Muiño da Veiga hot spring in the Leptospirae and Aquificae classes, respectively. Other MAGs were assigned to previously reported thermophilic genus: *Caldimicrobium* (MAG002), *Fervidobacterium* (MAG010), *Dictyoglomus* (MAG014) and *Calditerrivibrio* (MAG019). *Caldimicrobium* species are able to grow chemolithoautotrophically with hydrogen oxidation or with the disproportionation of sulfur compounds, including sulfur, thiosulphate or sulphite [87,88]. *Fervidobacterium nodosum* and *Dictyoglomus thermophilum* are biotechnologically interesting microorganisms for their thermostable enzymes, including *F. nodosum* cellulase Cel5A [89] and *D. thermophilum* xylanase XynB [90], endoglucanase Cel5H [91] and xylosidase *Dt*Xyl [92]. The only described *Calditerrivibrio* species is *Calditerrivibrio nitrodeducens* [93], which has nitrate as its only electron acceptor and ammonium as the end product. The Uncultivated Bacteria and Archaea (UBA) is a set of MAGs recovered from publicly available databases [94]. MAG026 is assigned to UBA11096 and represents an Aquificae isolate from a hydrocarbon metagenome that has yet to be assigned to a known genus, further reinforcing the novelty of the microbial diversity in the hot spring.

**Figure 6:**
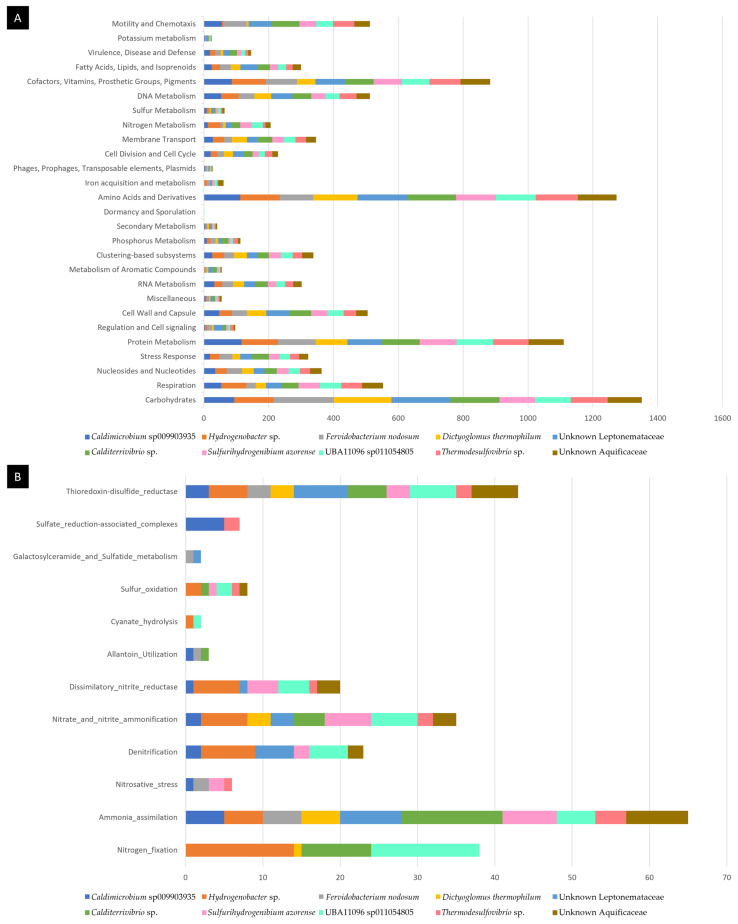
Functional annotation of MAGs. (**A**) Subsystems annotation using all classes that returned at least one gene count (not included the classes: Phages, Prophages, Transposable elements; Virulence; Photosynthesis). (**B**) Subsystems annotation using the categories under “Sulfur Metabolism” and “Nitrogen Metabolism”.

After taxonomical classification, MAGs were annotated using RASTtk. The total counts of genes annotated by their class in the Subsystems classification is provided in Figure 6A. A more detailed count for the genes involved in classes related to “Sulfur Metabolism” and “Nitrogen Metabolism” is provided in Figure 6B.

All MAGs had genes involved in ammonia assimilation and the ammonification of nitrate and nitrite, except for the *Fervidobacterium* MAG for the latter category. Nitrogen fixation genes were found mainly in the Aquificae MAGs for *Thermodesulfovibrio* MAG042 and UBA11096 MAG026 and in the *Calditerrivibrio* MAG019. Denitrification was found in all four Aquificae MAGs (*Hydrogenobacter*, *Sulfurihydrogenibium*, UBA11096 and the unknown Aquificaceae MAG043) as well as *Caldimicrobium* and the unknown Leptonemataceae MAG016. Nitrate reductase was found in all MAGs except for *Fervidobacterium*, *Dictyoglomus* and *Calditerrivibrio*. Only *Caldimicrobium* and *Thermodesulfovibrio* had sulfate reduction-associated complex genes, whereas sulfur oxidation was found in four *Aquificae* MAGs and in *Calditerrivibrio* and *Thermodesulfovibrio*. These results further confirm our previous analysis for the most abundant species found in the hot spring of Muiño da Veiga, notably because the *Sulfurihydrogenibium* MAG did contain the predicted denitrification and sulfur oxidation genes.

## 3. Materials and Methods

### 3.1. Metagenomic DNA Source: Site Description and Sample Collection

Water was collected from a hot spring source in the northwest of Spain, specifically the Muiño da Veiga hot spring (42°21′05.3″ N 7°54′36.3″ W) in the outskirts of Ourense, a city located in the Galicia region. A schematic representation of the sampling site is provided in Supplementary Figure S2. The water springs from a manually operated pump, and the site also consists in four hot-water pools open to the public next to the Miño river. The water was pumped several times before collection, and the date was December 2015.

Before sample collection, measurements of temperature and pH were made with a thermometer and pH strips. A total of 125 L of water (split in five 25 L bottles) were collected from the source in ethanol-washed plastic bottles to minimize microbial contamination and were washed several times on-site with the hot spring’s water before final sample collection. The samples were transported to the laboratory on the date of collection for processing in the shortest amount of time possible. The processing of samples, including the DNA extraction step, was performed within the same week as the collection of the samples, starting the next day after collection.

### 3.2. DNA Extraction

The protocol outlined in the commercial kit “Metagenomic DNA isolation kit for water” (Epicentre, Madison, WI, USA) was adopted with slight modifications in order to extract the DNA from the microorganisms present in the sample (the metagenomic DNA). This method is based on a combination of chemical and enzymatic treatments for the extraction, allowing the recovery of high molecular weight DNA randomly sheared in fragments of approximately 40 kb of length.

Water was filtered under a vacuum with a 0.2 μm membrane filter, and for every 25 L filtered, 2 to 4 filter membranes were obtained. Two of these filters were used in each extraction procedure. DNA was resuspended in 30 μL Tris Buffer 10mM, and extractions were pooled together.

### 3.3. DNA Sequencing and Sequence Analysis

The DNA was sequenced with an Illumina platform (Hi-seq) by the sequencing services provided by Health In Code (A Coruña, Spain). A single sequencing library was built from the extracted metagenomic DNA, and the sequencing reaction was performed following the standard protocol and using the components provided by the manufacturer. The sequencing resulted in two files in FastQ format (each containing a pair-ended read with the DNA sequence and its quality) with a mean length of 100 bp for each sequence and a total of 104 million sequences per file. These two files were subjected to two different bioinformatic pipelines, with some common early steps. The common steps comprised basic quality filtering of the FastQ format raw sequence files, and pair-ended read merging. Briefly, the first pipeline operated with the merged reads directly to avoid changes in read counts, to estimate the amount of gene functions with a potential industrial application in the metagenome by alignment to two different databases of annotated gene functions (a custom SEED subsystems database and the non-redundant NCBI database). The second pipeline also used merged reads, but this time, they were uploaded to a web-based metagenome analysis server (MG-RAST) [95] to align the sequences to a database of annotated taxonomic features, with the purpose of understanding the composition of the microbial community. This analysis server also allowed the study of KEGG pathways with respect to analyzing possible metabolic interactions of the microbial community by alignment to two different hierarchical function-based databases (KEGG Orthology and SEED Subsystems). A third independent pipeline using the Department of Energy Knowledge Database (KBase) genomic data analysis tool [96] was also implemented to perform Metagenome-Assembled Genomes (MAGs) analysis.

### 3.4. Quality Filtering

Sequence data were preprocessed with the prinseq-lite Perl script [97] in order to assess quality control. Sequences were first trimmed using a quality score threshold of 25 by both right and left ends of the sequences, with around 303 thousand sequences trimmed only by the left side (none were trimmed by the right side). A minimum length of 60bp was required to pass the next filter, removing a total of 2.6 million sequences. The following step involved the removal of sequences containing at least an unknown (N) basepair, resulting in the filtering of 24.5 million sequences. Sequence complexity was assessed by the entropy method (using a threshold of 70), and 1 million sequences were filtered. Lastly, sequence duplicates (including reverse complement duplicates) were also removed from the dataset, with a total of 7.8 million sequences removed. After quality processing, a total of 6.2 million sequences from the first file and 5.4 million sequences for the second file were left as singletons (its pair-ended read could not pass the filter). These sequences were not used in the next steps of the analysis. A total of 79.6 million sequences passed the quality filters (76.52%) and were used for bioinformatics analyses.

### 3.5. Read Merging

Sequences corresponding to a pair-ended read were merged using the PEAR software [98] running on default settings. PEAR successfully merged 54 million sequences (67.85%). Sequences that could not be merged were discarded as they would alter the number of reads generating each count, thus providing false data when aligned to a database if they were used in conjunction with merged sequences.

### 3.6. Alignment to the SEED Subsystems Database

A database from the SEED subsystems was obtained by clustering the subsystems with an identity of 98%, as published in a previous work [99]. This reduced database was used to align the merged pair-end reads using the open-source algorithm DIAMOND [100], with a setting for e-value of 0.00001. The results of the alignment were further processed to only keep the best hits per sequence (not only those with a unique identifier and the highest e-value but also keeping those with the same identifier and the same e-value) using a custom script.

### 3.7. Alignment to the NCBI nr Database

The nr database from NCBI was retrieved from its server (ftp://ftp.ncbi.nih.gov/blast/db/FASTA/nr.gz) (accessed on 28 September 2016) [101]. This database was used to align the merged pair-end reads using DIAMOND [100], with a setting for the e-value of 0.00001. As with the SEED subsystems database, only results of the alignment with the highest e-value were kept for each sequence.

### 3.8. Sequence Analysis on the MG-RAST Web-Based Server

Sequences were uploaded to the MG-RAST [95] web-based server, which automatically applies its own bioinformatic analysis pipeline to provide an overview of the metagenome. The pipeline options for the upload were as follows: dereplication “yes”, screening “*H. sapiens*, NCBI v36”, dynamic trimming “yes”, minimum quality “15” and maximum low quality basepairs “5”.

After the upload, a more in-depth analysis was performed in the analysis tab of the web server, using three different databases: RefSeq [102] for the taxonomic assessment of the reads, and KEGG Orthology (KO) [103] and SEED Subsystems [104] for hierarchical functional assignments and KEGG mapping. The analysis parameters were as follows: e-value “5”, %-ident “60”, length “15”, min. abundance “1” and method “representative hit”. The taxonomical categories at the order level were visualized using the KRONA web utility [105].

### 3.9. Sequence Analysis on the KBase Web-Based Server

For MAG analysis the raw sequences were uploaded to KBase [96]. The following steps were taken to obtain MAGs and then to perform the taxonomic assignment and the functional annotation: (i) read quality was assessed using FASTAQC [106]; (ii) low-quality read sequences were trimmed using Trimmomatic [107] with a sliding window value of 4 and minimum quality for the sliding windows set at 30; (iii) Low-Complexity reads with Prinseq were filtered out [97] with the filtering method “Entropy” and the threshold is set as 70; (iv) the read quality was assessed once again using FASTQC; (v) metagenomic reads were assembled into contigs using the metaSPADES assembler [108]; (vi) the quality of the assemblies was assessed using QUAST [109]; (vii) contigs were binned using MetaBAT2 [110]; (viii) bins were filtered by completeness (more than 95%) and contamination (less than 2%) by comparison to a genome database using the quality assessment tool CheckM [111]; (ix) the taxonomic assignment of the MAGs was performed using Genome Taxonomy Database Toolkit (GTDB-Tk) [112,113,114,115,116,117,118,119]; (x) the functional annotation of the MAGs was performed using Rapid Annotations using the Subsystems Technology Toolkit (RASTtk) [120,121,122].

## 4. Conclusions

Considering all putative genetic and functional diversities found in the metagenome, a complex network of potential metabolic interactions within the microbial community can be established, where most elements cycles were found to be present. This diversity probably is the result of the optimization between using all possible nutrients and filling all available niches while minimizing metabolic overlaps, which is not unlike what has been described for other hot springs [9]. Other contributing factors include water temperatures that are not high enough to be a limiting diversity factor [123] and a circumneutral pH [124]. A synergetic effect has been described between metabolic diversity and the increase in biomass in the hot spring ecosystem observed when temperatures are below the hyperthermophilic range. The presence of varied primary producers with autotrophic pathways expands the range of available nutrients, and with the increase in biomass microbial mats, they become thicker and, in turn, expand the available niches [125]. Such niche-facilitating microbial mats have been described to be formed by the most abundant genus of the community, *Sulfurihydrogenibium* [11,126]. Up to 10 MAGs were obtained and 3 of them revealed putative novel diversity in the *Aquificae* and *Leptospirae* classes. Yet another three MAGs were assigned to microorganisms in the most abundant genera of the hot spring, including *Sulfurihydrogenibium*. Their functional annotation further confirmed predicted metabolic interactions as key members of the hot spring microbial community. Lastly, the presence of enzymes with possible biotechnological applications has been confirmed, both by the presence of two MAGs of biotechnologically relevant species and by functional database searches in the metagenome, making the hot springs of Muiño da Veiga interesting not only as a thermal site but as a source for thermozymes.

## Figures and Tables

**Table 1:** GTDB-Tk taxonomic assignment of MAGs.

MAG	Domain	Phylum	Class	Order	Family	Genus	Species	Method
002	Bacteria	Desulfobacterota	Thermodesulfobacteria	Thermodesulfobacteriales	Thermodesulfobacteriaceae	*Caldimicrobium*	*Caldimicrobium* sp009903935	topology and ANI
008	Bacteria	Aquificota	Aquificae	Aquificales	Aquificaceae	*Hydrogenobacter*	-	topology and ANI
010	Bacteria	Thermotogota	Thermotogae	Thermotogales	Fervidobacteriaceae	*Fervidobacterium*	*Fervidobacterium nodosum*	topology and ANI
014	Bacteria	Dictyoglomota	Dictyoglomia	Dictyoglomales	Dictyoglomaceae	*Dictyoglomus*	*Dictyoglomus thermophilum*	topology and ANI
016	Bacteria	Spirochaetota	Leptospirae	Leptospirales	Leptonemataceae	-	-	RED
019	Bacteria	Deferribacterota	Deferribacteres	Deferribacterales	Calditerrivibrionaceae	*Calditerrivibrio*	-	RED
023	Bacteria	Aquificota	Aquificae	Hydrogenothermales	Hydrogenothermaceae	*Sulfurihydrogenibium*	*Sulfurihydrogenibium azorense*	topology and ANI
026	Bacteria	Aquificota	Aquificae	Aquificales	Aquificaceae	UBA11096	UBA11096 sp011054805	topology and ANI
042	Bacteria	Nitrospirota	Thermodesulfovibrionia	Thermodesulfovibrionales	Thermodesulfovibrionaceae	*Thermodesulfovibrio*	-	topology and ANI
043	Bacteria	Aquificota	Aquificae	Aquificales	Aquificaceae	-	-	topology

## Data Availability

The dataset of the metagenome supporting the conclusions of this article is available in the MG-RAST repository: https://www.mg-rast.org/linkin.cgi?project=mgp84238 (accessed on 3 October 2022).

## Supplementary Materials

The following supporting information can be downloaded at: https://www.mdpi.com/article/10.3390/ijms232012241/s1.
