# Supplementary material for: Insights on Microbial Communities Inhabiting Non-Volcanic Hot Springs
Source: Int J Mol Sci. 2022 Oct 13;23(20):12241. doi: 10.3390/ijms232012241 (PMC9603410; doi:10.3390/ijms232012241)
Supplement: Supplementary file 1 [file ijms-23-12241-s001.zip › ijms-1825021-supplementary.pdf]

International Journal of
Molecular Sciences

Article

Insights on Microbial Communities Inhabiting Non-Volcanic Hot Springs

Juan-José Escuder-Rodríguez, María-Eugenia DeCastro, Almudena Saavedra-Bouza, Manuel Becerra * and María-Isabel González-Siso *

Grupo EXPRELA, Centro de Investigacións Científicas Avanzadas (CICA), Departamento de Bioloxía, Facultade de Ciencias, Universidade da Coruña, 15071 A Coruña, Spain
* Correspondence: manuel.becerra@udc.es (M.B.); isabel.gsiso@udc.es (M.-I.G.-S.)

Supplementary Materials: The following supporting information can be downloaded at: www.mdpi.com/xxx/s1, Figure S1: Schematic representation of Muiño da Veiga hot springs and sampling site. Figure S2: Rarefaction plot. Figure S3: CheckM plot. Table S1: Statistics for the metagenome from Muiño da Veiga hot spring. Table S2. CheckM report.

Citation: Escuder-Rodríguez, J.-J.; DeCastro, M.-E.; Saavedra-Bouza, A.; Becerra, M.; González-Siso, M.-I. Insights on Microbial Communities Inhabiting Non-Volcanic Hot Springs. *Int. J. Mol. Sci.* **2022**, *23*, 12241. https://doi.org/10.3390/ijms232012241

Academic Editors: Salvatore Fusco and Bettina Siebers

Received: 4 July 2022
Accepted: 4 October 2022
Published: 13 October 2022

Publisher's Note: MDPI stays neutral with regard to jurisdictional claims in published maps and institutional affiliations.

Copyright: © 2022 by the authors. Submitted for possible open access publication under the terms and conditions of the Creative Commons Attribution (CC BY) license (https://creativecommons.org/licenses/by/4.0/).

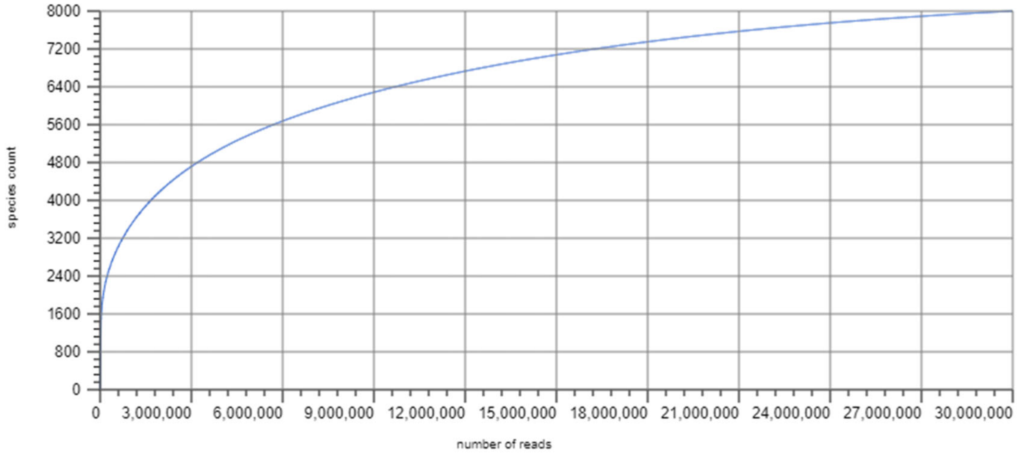

Supplementary Figure S1. Rarefaction plot. Assessment of the sequencing depth by plotting species annotation counts against the number of reads analyzed.

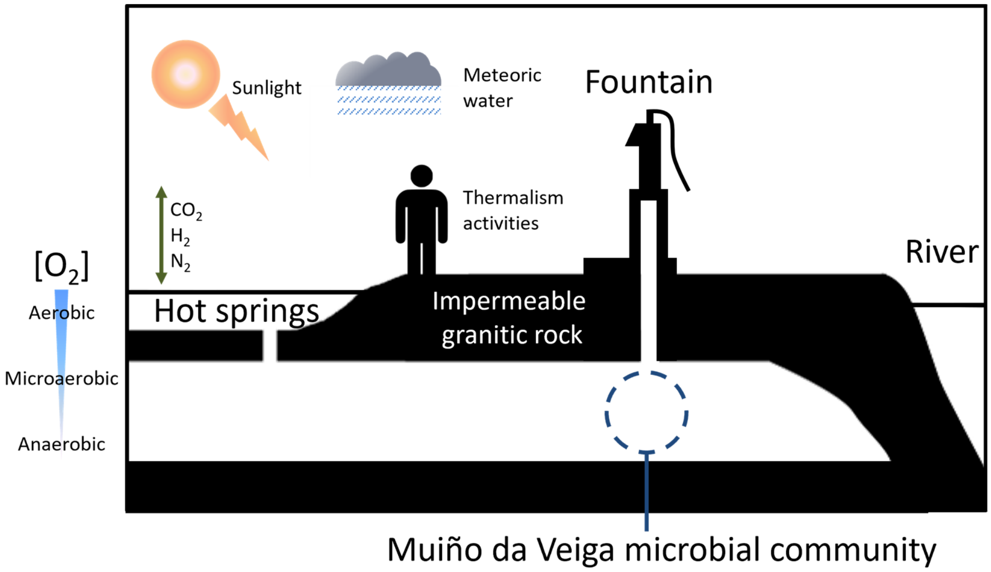

Supplementary Figure S2. Schematic representation of Muiño da Veiga hot springs and sampling site. The impermeable granitic rock prevents water with meteoric origins (rain, Miño river) from mixing with the subterranean hot water. Sunlight exposition is limited to the surface of the hot spring's pools, where the exchange of gaseous elements takes place. Oxygen concentration decreases with water depth. As the pools are open for thermalism activities, some degree of cross contamination may take place too.

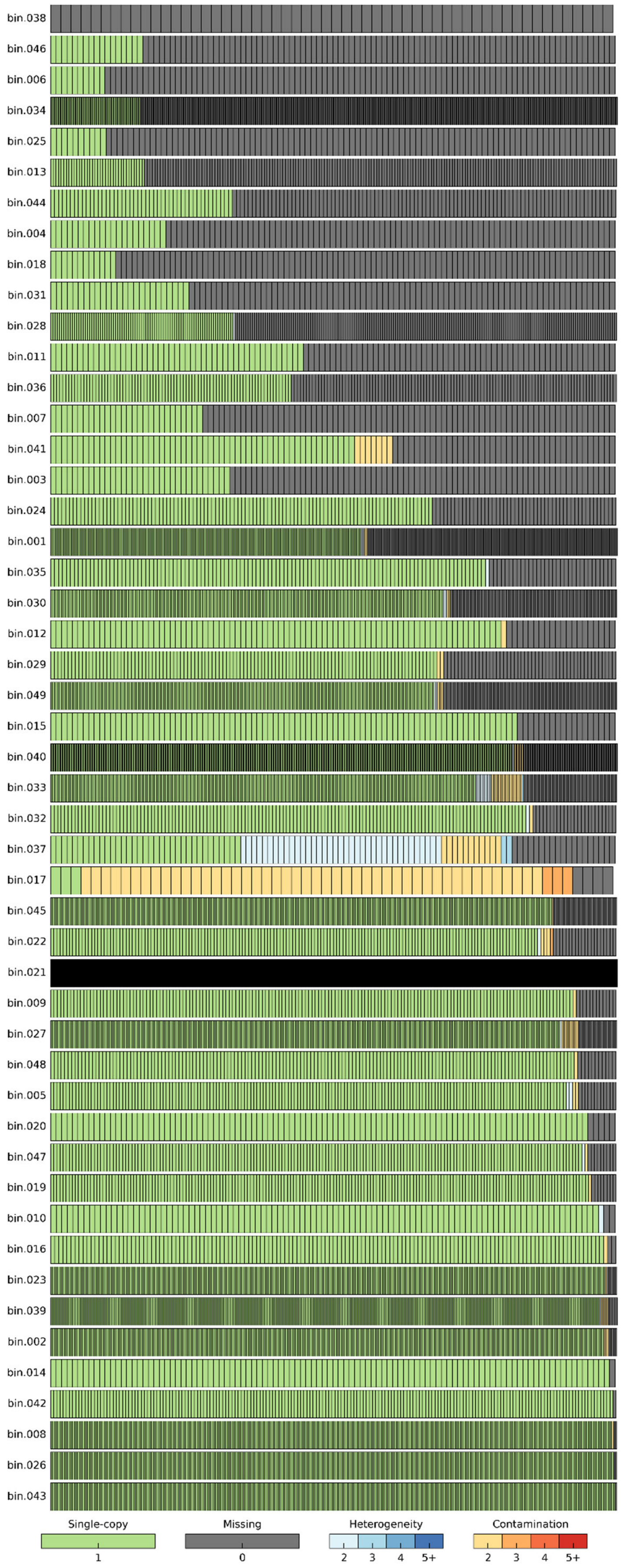
bin.038
bin.046
bin.006
bin.034
bin.025
bin.013
bin.044
bin.004
bin.018
bin.031
bin.028
bin.011
bin.036
bin.007
bin.041
bin.003
bin.024
bin.001
bin.035
bin.030
bin.012
bin.029
bin.049
bin.015
bin.040
bin.033
bin.032
bin.037
bin.017
bin.045
bin.022
bin.021
bin.009
bin.027
bin.048
bin.005
bin.020
bin.047
bin.019
bin.010
bin.016
bin.023
bin.039
bin.002
bin.014
bin.042
bin.008
bin.026
bin.043
Single-copy
1
Missing
0
Heterogeneity
2 3 4 5+
Contamination
2 3 4 5+

Supplementary Figure S3. CheckM plot of the bins obtained from Muiño da Veiga metagenomic read processing. Single copy phylogenetic markers are used to evaluate the bin's completeness and contamination: green color indicates one copy present for the marker gene in the bin; gray indicates an absence; the various shades of orange indicate multiple copies of the marker gene, suggesting a contamination of the bin; various shades of blue indicate multiple copies of the marker gene from closely related strains, suggesting strain heterogeneity in the sample.

Supplementary Table S1. Statistics for the metagenome from Muiño da Veiga hot spring.

FEATURE	NUMBER	UNITS
Sequences (uploaded)	27,113,937	sequences
Basepairs (uploaded)	3,968,584,153	bp
Mean sequence length (uploaded)	146 ± 24	bp
Mean GC content (uploaded)	44 ± 13	%
QC failed	9,290,572<br>34.265	Sequences<br>% of total
Artificial duplicate reads	4,807,111<br>17.729	Sequences<br>% of total
Sequences (post QC)	17,823,365<br>65.735	Sequences<br>% of total
Basepairs (post QC)	2,658,836,275<br>66.997	bp<br>% of total
Mean sequence length (post QC)	149 ± 24	bp
Mean GC content (post QC)	46 ± 14	%
Predicted features	16,639,799<br>93.359	sequences<br>% of total post QC
rRNA genes	72,722<br>0.408	sequences<br>% of total post QC
Predicted protein with known function	13,052,391<br>73.232<br>78.441	sequences<br>% of total post QC<br>% of predicted features
Predicted protein with unknown function	3,514,686<br>19.720<br>21.122	sequences<br>% of total post QC<br>% of predicted features
Unknown features	1,183,566<br>6.641	sequences<br>% of total post QC

Supplementary Table S2. CheckM report for Muiño da Veiga hot spring bins.

BIN	# Genomes	# Markers	# Marker Sets	0	1	2	3	Completeness	Contamination
001	193	427	214	188	234	5	0	58.19	1.67
002	138	335	243	5	327	3	0	97.94	1.23
003	5449	104	58	71	33	0	0	48.28	0.0
004	5449	103	57	82	21	0	0	18.66	0.0
005	2258	181	110	12	165	4	0	92.67	1.82
006	5449	104	58	94	10	0	0	13.79	0.0
007	5449	104	58	76	28	0	0	41.38	0.0
008	138	336	246	2	333	1	0	99.19	0.1
009	901	171	117	12	158	1	0	91.03	0.85
010	5443	102	57	2	99	1	0	96.49	1.75
011	5443	105	59	58	47	0	0	32.9	0.0
012	5449	104	58	20	83	1	0	68.39	1.72
013	54	217	168	181	36	0	0	17.26	0.0
014	5449	104	58	1	103	0	0	98.28	0.0
015	5449	104	58	18	86	0	0	73.41	0.0
016	2993	140	85	2	137	1	0	97.65	1.18
017	5656	56	24	4	3	46	3	86.11	78.7
018	5449	104	58	92	12	0	0	18.97	0.0
019	2258	184	114	8	175	1	0	95.95	0.88
020	5449	104	58	5	99	0	0	92.95	0.0
021	134	1169	335	146	1012	11	0	87.87	0.72
022	2258	180	109	20	155	4	1	87.36	3.26
023	138	338	246	6	331	1	0	97.76	0.2
024	924	151	101	49	102	0	0	57.72	0.0
025	5449	102	57	92	10	0	0	15.79	0.0

026	138	336	246	2	334	0	0	99.19	0.0
027	138	338	246	23	304	11	0	91.4	3.05
028	1495	261	164	176	84	1	0	29.19	0.61
029	924	161	108	49	110	2	0	70.58	1.23
030	169	313	233	92	217	4	0	68.31	1.07
031	5449	102	57	77	25	0	0	27.19	0.0
032	924	163	110	24	137	2	0	83.18	1.82
033	169	316	236	52	237	26	1	82.51	7.55
034	32	488	209	411	77	0	0	15.03	0.0
035	2993	143	89	32	110	1	0	66.56	0.1
036	2258	188	117	108	80	0	0	35.16	0.0
037	5449	104	58	19	35	48	2	84.97	49.06
038	5656	56	24	56	0	0	0	0.0	0.0
039	323	386	233	6	374	6	0	97.85	2.15
040	30	540	241	90	439	11	0	80.03	1.28
041	5449	104	58	41	56	7	0	43.97	4.23
042	2258	181	110	1	180	0	0	99.09	0.0
043	138	336	246	1	335	0	0	99.59	0.0
044	2993	143	89	97	46	0	0	17.59	0.0
045	138	338	246	38	299	1	0	86.59	0.2
046	5449	104	58	87	17	0	0	13.17	0.0
047	2258	181	110	9	170	2	0	93.18	1.82
048	924	163	110	11	151	1	0	91.82	0.91
049	169	316	236	97	214	5	0	72.23	1.91
